# Status Epilepticus: Behavioral and Electroencephalography Seizure Correlates in Kainate Experimental Models

**DOI:** 10.3389/fneur.2018.00007

**Published:** 2018-01-23

**Authors:** Shaunik Sharma, Sreekanth Puttachary, Achala Thippeswamy, Anumantha G. Kanthasamy, Thimmasettappa Thippeswamy

**Affiliations:** ^1^Epilepsy Research Laboratory, Department of Biomedical Sciences, College of Veterinary Medicine, Iowa State University, Ames, IA, United States; ^2^Veterinary Biomedical Sciences, College of Veterinary Medicine, Oregon State University, Corvallis, OR, United States; ^3^Parkinson’s Research Laboratory, Department of Biomedical Sciences, College of Veterinary Medicine, Iowa State University, Ames, IA, United States

**Keywords:** status epilepticus, kainate, repeated low dose, electroencephalography seizures, behavioral seizures

## Abstract

Various etiological factors, such as head injury, chemical intoxication, tumors, and gene mutation, can induce epileptogenesis. In animal models, *status epilepticus* (SE) triggers epileptogenesis. In humans, convulsive SE for >30 min can be a life-threatening medical emergency. The duration and severity of convulsive SE are highly variable in chemoconvulsant animal models. A continuous video-electroencephalography (EEG) recording, and/or diligent direct observation, facilitates quantification of exact duration of different stages of convulsive seizures (Racine stages 3–5) to determine the severity of SE. A continuous convulsive SE for >30 min usually causes high mortality in some rodents and results in widespread brain damage in the surviving animals, in spite of treating with antiepileptic drugs (AEDs). AEDs control behavioral seizures but not EEG seizures. The severity of initial SE impacts epileptogenesis and cognitive function; therefore, quantitative assessment of behavioral SE and EEG in animal models will help to understand the impact of SE severity on epileptogenesis. There are several excellent reviews on experimental models of seizure/SE/epilepsy. This review focusses on the comparison of induction and characterization of behavioral SE and EEG correlates in mice and rats induced by kainate. We also discuss the advantages of repeated low dose of kainate (i.p. route), which minimizes variability in the initial SE severity between animals and reduces mortality rate. A refined approach to induce SE with kainate also addresses the two of the 3Rs (i.e., refinement and reduction), the guiding principles for ethical and scientific standpoint of animal research.

## Introduction

The PubMed search, using *status epilepticus* (SE) and epilepsy as key words, yielded >1,600 articles on SE. In a recent review on bibliometric analysis of top 100 articles on both SE and epilepsy, as expected, revealed that the majority of these articles are from animal studies (1). In these literature, the duration of SE is discussed, but the information on quantification of the severity of SE is lacking (2, 3). In general, the duration of SE in experimental models is between the time of administration of chemoconvulsant (assuming the onset of convulsive SE) and the end of behavioral seizures. Traditionally, the behavioral seizures are scored based on the Racine scale (4–6). However, the electroencephalography (EEG) quantification to determine the severity of SE in animal models is lacking in the literature. There are numerous reports on qualitative EEG that has been used as an evidence to demonstrate the occurrence of SE and to support the idea that benzodiazepines are ineffective in controlling the epileptiform spiking activity after certain time-point (7, 8). We have recently described both behavioral and EEG seizures’ quantification methods from the kainate-induced SE in both mice and rat models (2, 9, 10). In this mini-review, we discuss SE in the context of its onset, duration, types of seizures during SE, quantitative measures of behavioral and electrographic seizures to determine SE severity, and the impact of SE severity on epileptogenesis in the rat and mouse kainate models of temporal lobe epilepsy (TLE).

The definition of SE has been constantly changing. It is also suggested that it depends on the context of SE, in humans or experimental models (11, 12). There has been a consensus that the duration or length of convulsive seizures (CS) during SE should be sufficient to cause long-term brain injury, enabling the brain to generate spontaneous seizures i.e., “an enduring epilepticus” (13). According to the Commission of Epidemiology Prognosis (1993) and Dodson et al. (14), initial duration of SE for humans was set at 60 min. This was later reduced to 30 min, which is now widely accepted for studies that investigate the long-term consequences of SE, i.e., epileptogenesis and epilepsy. Seizures are normally self-terminated by activating inhibitory mechanisms. Failure of these mechanisms can result in prolonged seizures (SE), which may require interventional drugs (12). However, according to the clinical trial guidelines, the treatment should be initiated only if the CS last for ~5 min (11, 14–16). According to the recommendation of the Commission on Classification and Terminology, and the Commission on Epidemiology of the International League Against Epilepsy (ILAE), the SE is defined as “*a condition resulting either from the failure of the mechanisms responsible for seizure termination or from the initiation of mechanisms, which lead to abnormally, prolonged seizures (after time point t1). It is a condition, which can have long-term consequences (after time point t2), including neuronal death, neuronal injury, and alteration of neuronal networks, depending on the type and duration of seizures*” (17). In this review, we discuss the types and duration of seizures during SE in mice and rat kainate models of TLE. We also discuss SE in these models on the background of a new definition of SE proposed by the ILAE.

## SE in Animal Models: The Types of Seizures

**Figure 1:**
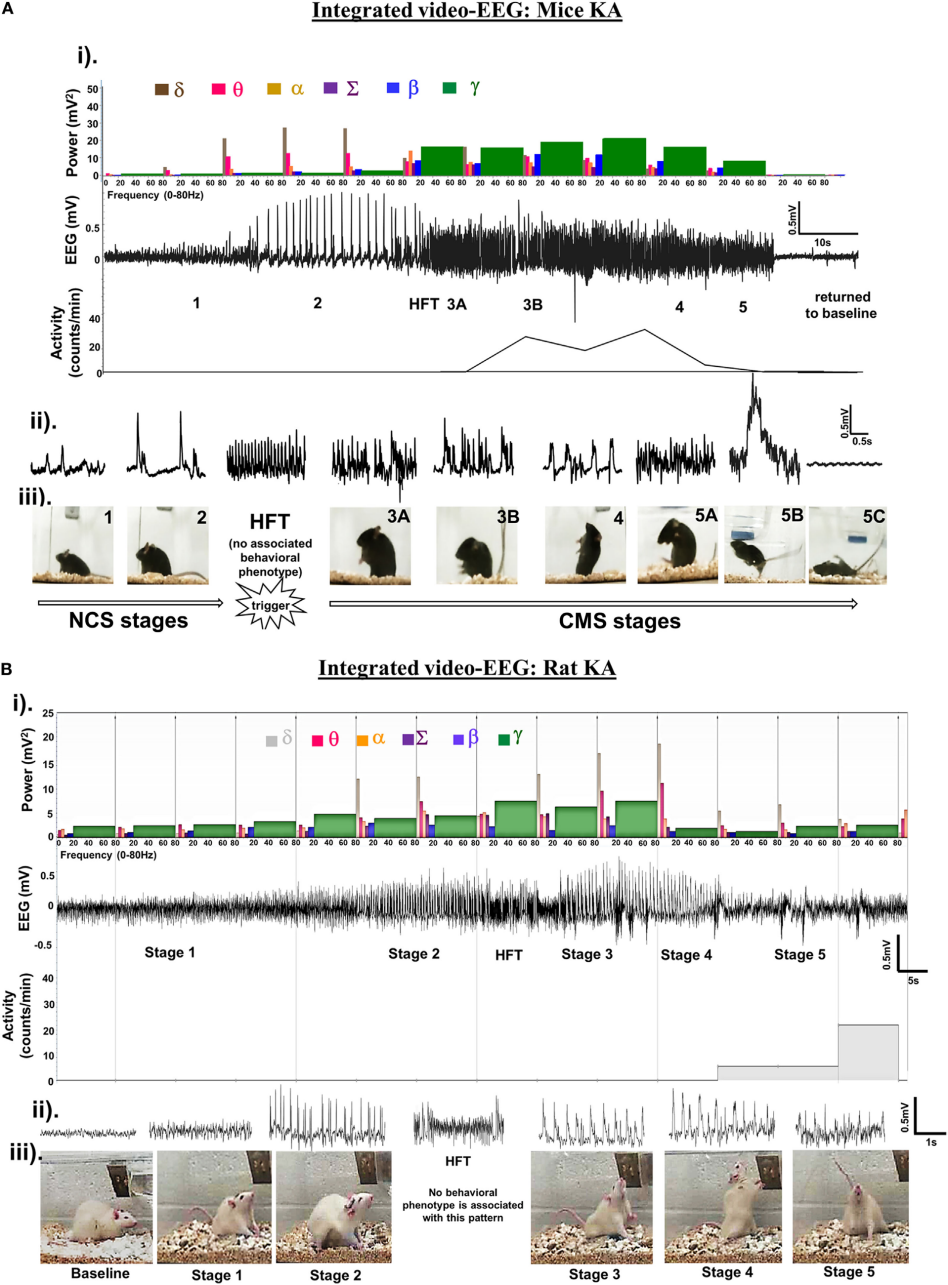

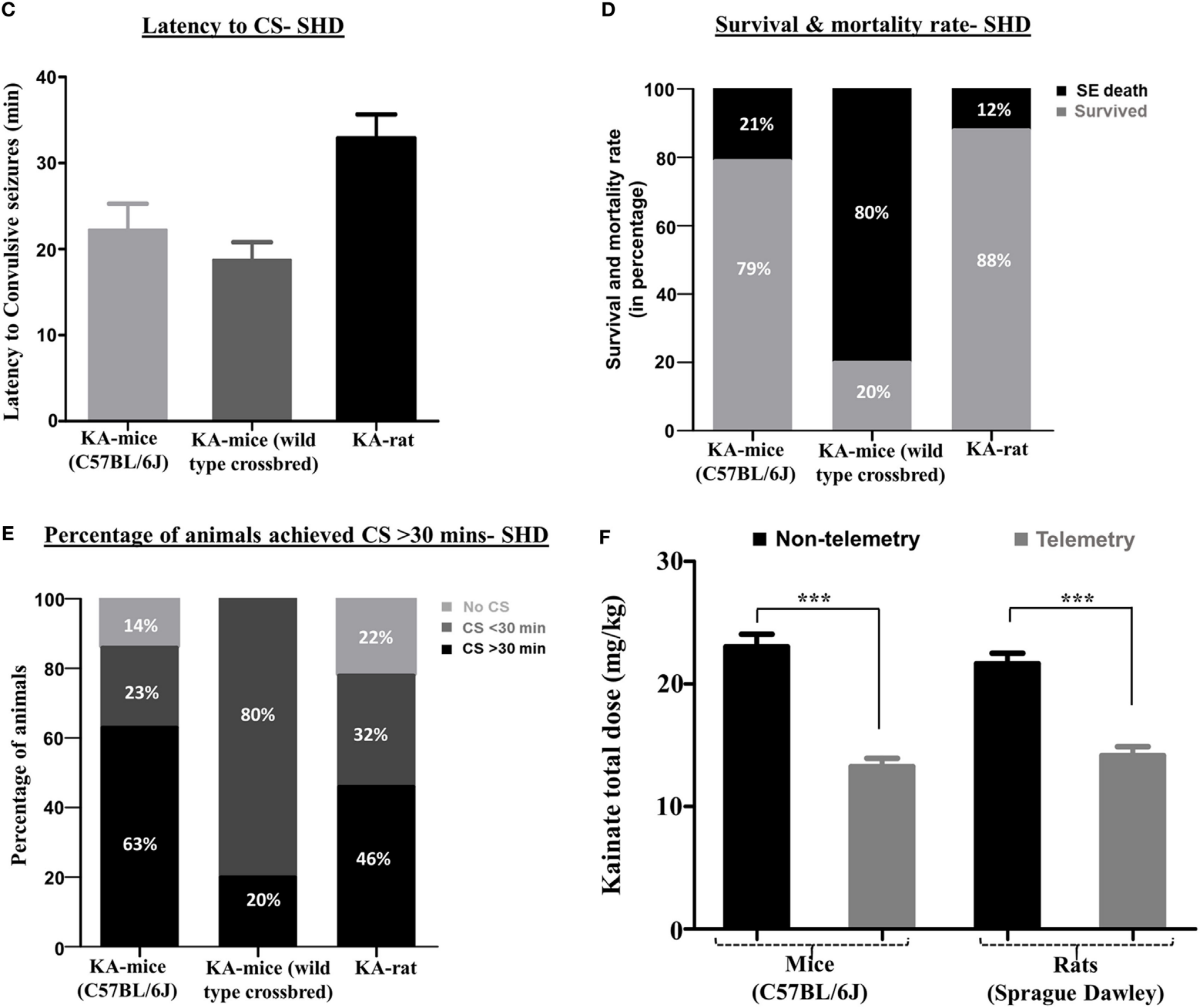
**(A,B)** An example of an electroencephalography (EEG) trace showing different stages of seizures, induced by kainate in C57BL/6J mouse **(A)** and Sprague Dawley rat **(B)**. The corresponding power spectra, activity level, and real-time behavioral seizures captured from the video-EEG recording are illustrated. (i) EEG trace in the middle shows the changes in electrical activity as the seizure severity progressed from NCS to CS over time. A brief HFT pattern on the EEG, which had no behavioral correlate, preceded the CS. The photographs show different stages of behavioral seizures (iii). Magnified 2-s EEG traces that correspond to each stage of behavioral seizures are shown in the panel (ii). The histograms at the top panel in “i” represent power bands. As the seizure progressed from NCS to CS, the power bands, especially the gamma power increased (shown in green) at stage 3, but decreased during stages 4 and 5. The delta and theta power bands increased during the stage 2 NCS. The gamma power band increased after HFT, peaked at stage 3B, and declined in stages 4 and 5 before returning to the baseline. One interesting finding in the power spectra of the rat kainate model **(B)**, in contrast to the mouse kainate model **(A)**, was an increase in theta and delta power bands during CS. Activity counts represent brisk locomotor activity, which is shown below the EEG trace, increased from stage 3A onward and peaked in stage 3B and 4. Activity counts reduced in stage 5 when the mouse was recumbent or showed generalized rigidity but increased when the mouse displayed jumping behavior. In the rat KA model, the delta power persisted at high levels during pre- and post-CS stage, but the theta, beta, and gamma powers increased during the CS stages. **(C–E)** The comparison of latency to the onset of CS **(C)**, mortality rate **(D)**, and the time spent by the number of animals (in percentage) in CS stages **(E)** in C57BL/6J mice, the crossbred mice on C57BL/6J genetic background (C57 × Balb-c) and the Sprague Dawley rats in response to SHD of kainate. **(F)** Comparison between the total amounts of kainate, given 5 mg/kg at 30 min interval, required to induce CS in telemetry and non-telemetry groups of mice and rats. The animals with telemetry required about ~40% less kainate to reach CS when compared to the non-telemetry group in both rats and mice. Mann–Whitney test, ***p < 0.001, *n* = 30–40 per group. RLD, repeated low dose; SHD, single high dose; CS, convulsive seizures; NCS, non-convulsive seizures; HFT, high frequency trigger. Adopted and modified from Tse et al. (2) and Puttachary et al. (9).

In the literature on chemoconvulsant models of seizure/epilepsy, the types of behavioral seizures during SE are generally well defined based on the (modified) Racine scale (4–6, 18). However, the scoring pattern varies between the literature, models, stimulus, strains, and species used in the experiment. The most acceptable broad classification of behavioral seizures, for quantification purpose, are non-convulsive seizure (NCS) and CS (tonic–clonic, tonic, and clonic). For the sake of clarity, the following five stages of behavioral seizures were considered for SE quantification for mice and rat kainate models: stage 1, absence-like immobility; stage 2, hunching with facial automatism (exaggerated upper lip movement as evident from vibrissae movement with or without salivation) and/or abducted forelimb/s, wet-dog shaking (in rats); stage 3, rearing with facial automatism and forelimb clonus (excessive salivation in some animals); stage 4, repeated rearing with continuous forelimb clonus and falling (loss of righting reflex); and stage 5, generalized tonic–clonic convulsions with lateral recumbence or jumping (more common in mice) and wild running followed by generalized convulsions (Figures 1A,B) (2, 9).

## SE Onset and Its Length or Duration in Animal Models

**Figure 2:**
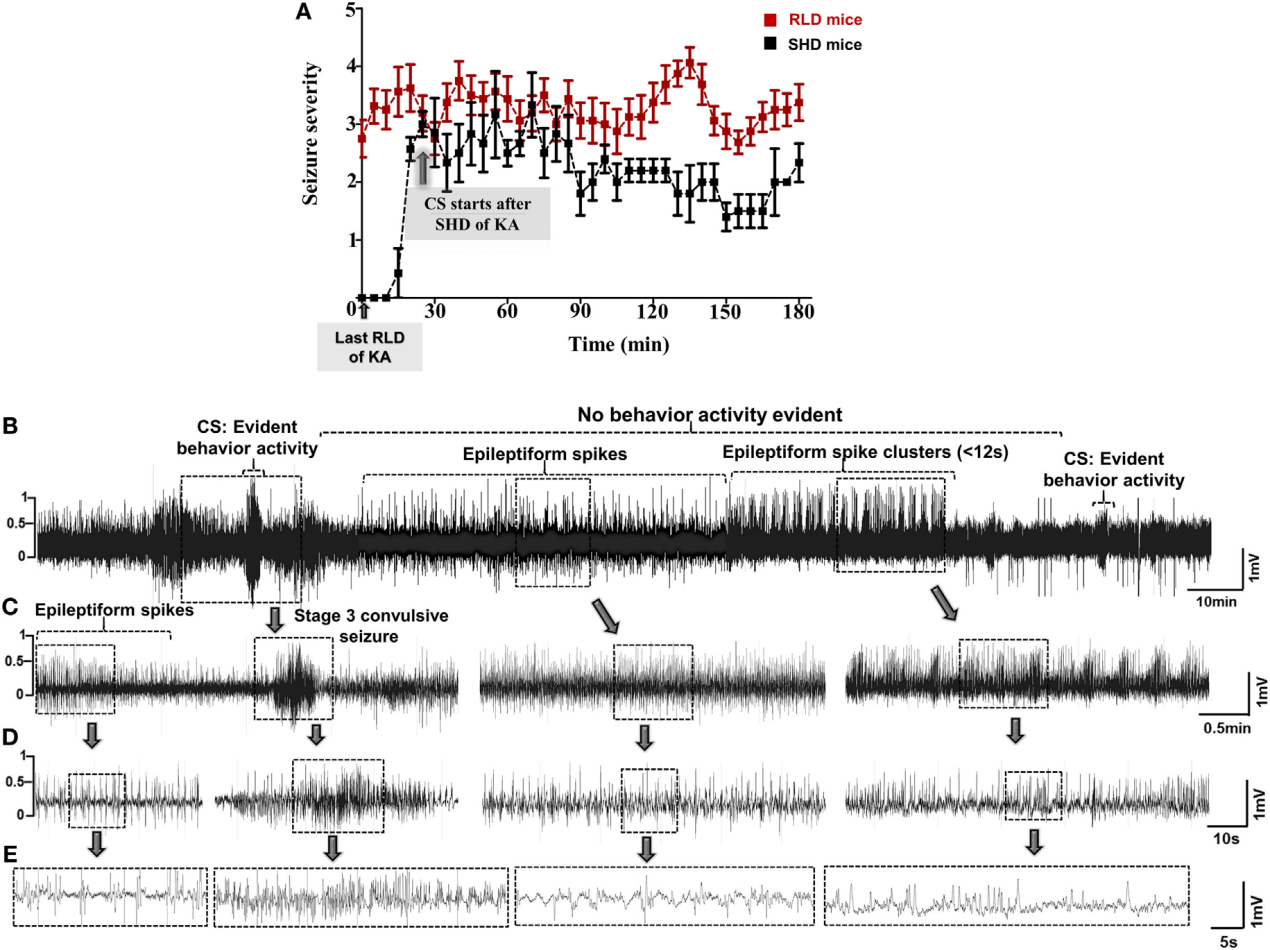
**(A)** Seizure severity comparison between single high dose (SHD) and repeated low dose (RLD) of KA in mice during SE. Animals in RLD group showed more severe seizures and spent more time in ≥3 stage when compared to the SHD group during the 3 h of SE. Animals in both groups were given 20–30 mg/kg kainate (i.p.) (***p < 0.0001, two-way ANOVA between 1 and 719 degrees of freedom, *F* = 148.60, *n* = 30 for each group). Adopted and modified from Tse et al. (2). **(B–E)** An example of a 30-min EEG trace, during SE, showing inter-ictal activity that comprises of continuous epileptiform spikes and seizure cluster <12 s without any behavioral phenotype. Convulsive seizures (CS) and non-convulsive seizures (NCS) were observed throughout SE, but behavior activity was evident only during CS, i.e., stage ≥3. Expanded electroencephalography (EEG) traces of **(B)** are shown in panels **(C–E)**.

Unlike electroconvulsant models, such as the maximal electroshock seizure model (19–21), the time of onset and length of convulsive SE are highly variable in chemoconvulsant models. This depends on numerous factors, such as drug and its route of administration, species, sex, age groups, strain, and genetic background (22–25). In the kainate model, intraperitoneal administration of a single high dose (SHD) of kainate (20–30 mg/kg) induced convulsive SE between 5 and 49 min in 86% of inbred C57BL/6J mice. Out of these, 63% of mice had >30 min and 23% had <30 min of convulsive SE (Figure 1E). The duration of SE (stages 1–5) was usually greater than 2 h in these animals (Figure 2A). However, 21% of mice died due to the severity of CS, and the vast majority of deaths occurred in mice that reached stage 5 seizures in <15 min of kainate administration (data not shown). A similar pattern of quick onset of convulsive SE with kainate (i.p.), but >80% mortality was observed in mixed genetic background mice (C57 × Balb-c) (Figures 1C–E). In Sprague Dawley rats, the convulsive SE onset with a SHD of kainate (12.5–15.0 mg/kg, i.p.) occurred between 30 and 40 min, with 12% mortality, and the percentage of animals that achieved CS for ≥30 min was 46% (Figures 1C–E).

The type of seizures and their duration determine the severity of SE. It has been proposed that >10 min of convulsive SE is sufficient to cause brain damage and to induce TLE in chemoconvulsant animal models (10, 26). However, in some of the literature, there is limited information on whether the CS (stage ≥3) were continuous or intermittent in a given time (for example, during the 2–3 h of drug administration), and whether the behavioral SE was terminated with an antiepileptic drug (AED) or not. We used both behavioral methods and EEG parameters to accurately quantify the duration of convulsive SE. Interestingly, in C57BL/6J mouse kainate model, about 50% of behavioral seizures did not correspond to the epileptiform activity detected on the EEG (10). This implies that in some chemoconvulsant models, there is an exaggerated behavioral response that could be due to the peripheral effects (27).

## Quantitative Measure of Behavioral and Electrographic Seizures to Determine SE Severity

In a pilocarpine mouse model (C57BL/129Sv genetic background) of acute seizures, both video and quantitative EEG analyses (fast Fourier transformation) were used to correlate the electrographic seizures with the convulsive behaviors (6). In this study, the authors graded the seizures on the Racine scale, and the root-mean-square (RMS) power analysis of EEG was performed using Sirenia Seizure Pro software. They found a weak correlation between the RMS power and convulsive behavior, induced by the pilocarpine, suggesting that power spectral analysis alone is insufficient to quantitatively correlate the behavioral seizures with electrographic seizures (6). In the mouse (C57BL/6J) kainate model, we determined the power spectrum for frequencies ranging from 0.5 to 80 Hz based on stage-specific behavioral and EEG seizure correlates (2). In our studies, the power band spectrum analysis included delta (δ, 0–4 Hz), theta (θ, 4–8 Hz), alpha (α, 8–12 Hz), sigma (Σ, 12–16 Hz), beta (β, 16–24 Hz), and gamma (γ, 24–80 Hz) frequencies in 10-s epochs on a continuous EEG during SE with power ranging from 200 to 2,000 mV^2^. As the seizure stages progressed from stages 1–5, the EEG patterns with increase in power bands began to emerge in real-time as measured by the Neuroscore software (Figures 1A,B). A magnified EEG traces shown above the behavioral stages in these figures demonstrate stage-specific spike characteristics (amplitude, duration, and inter-spike intervals). When the seizures progressed from NCS (stages 1 and 2) to CS (stages 3–5), the power in different bands also changed. The gamma power peaked during stage 3, but declined in stages 4 and 5 before reaching the baseline (Figures 1A,B). Higher delta and theta powers were the hallmarks of stage-2 spikes. A reduced delta power, but the increased beta and gamma powers marked the progression from NCS to CS. The high frequency trigger (HFT) pattern was frequently observed during the transition from NCS to CS, which was characterized by a brief peak in alpha and sigma powers (Figures 1A,B). To determine the severity of SE, in addition to the power spectral analysis, it is useful to quantify stage-specific spike frequencies during SE to correlate with the behavioral seizures as described in the mouse and rat kainate models (2, 9, 10). However, it is plausible that the type of chemoconvulsant [for example, parasympathomimetics (pilocarpine) versus glutamate agonists (kainate)] could affect the extent of correlation between the power, EEG, and behavioral seizures.

## Severity of SE and Its Impact on Epileptogenesis

Several review articles have documented the impact of the severity of initial SE on epileptogenesis and epilepsy in the long term (28–32). The majority of chemoconvulsant-induced SE in rats and mice will develop progressive epilepsy, which is generally characterized by reactive gliosis, neurodegeneration, spontaneous recurrent seizures, and cognitive deficits (10, 31–37). A recent article by Loscher et al. (22) has highlighted the relevance of strain-specific differences in mice and rats and their implications in determining the choice of experimental models of seizure/epilepsy. The vast variation in seizure susceptibility in animal models is due to the diversity of their outbred genetic backgrounds. However, the C57BL/6J inbred mice response to chemoconvulsants such as kainate also varies between batches of mice and the source (22, 38–41). SE induction by chemoconvulsants in mice and rats revealed a huge variation in latency to convulsive SE onset, duration of CS, and mortality (Figures 1C–E and 2A). A refined method of inducing SE with repeated low dose (RLD) of kainate, 5 mg/kg, administered at 30 min intervals *via* the intraperitoneal route, has shown to reduce inconsistency in SE severity across different strains (2, 42–44). Moreover, the RLD method of kainate administration revealed that the surgical procedure for intracranial electrode implantation reduces the threshold for CS onset. About 40% less kainate was required to induce convulsive SE in the telemetry animals when compared to the non-telemetry animals (Figure 1F) (2, 9, 10). The SE induction by either SHD or RLD of kainate (i.p.) will lead to the development of epilepsy in the majority of animals. However, the frequency of spontaneous convulsive (tonic–clonic) seizures significantly varies between models. For example, the RLD method in both rats and mice induced epilepsy and the C57BL/6J mice had almost the same numbers of spontaneous CS as the rats during the first 4 weeks, but the frequency of seizures reduced after 4–6 weeks in the mice (9, 10, 45). In both rats and mice, the electrodes were implanted epidurally on the surface of the cortices as described previously (2, 10, 46). Interestingly, in a 4-month continuous video-EEG study from the C57BL/6J mouse kainate model, irrespective of initial severity of SE (severe or mild SE), high numbers of electrographic NCS were detected on EEG, which persisted during entire length of the study (10). However, the frequency of spontaneous CS, a readout for the classification of the severity of disease, was related to the initial severity of SE in both rats and mice (9, 10, 47, 48). Therefore, it is imperative to review and refine the methods of induction and quantification of severity of SE (the type and duration of seizures) to understand its impact on the disease progression and, importantly, to determine disease-modifying effects of drugs in experimental models of TLE.

Considering one of the operational dimensions of the ILAE recommendations for SE, we further focus the review on “*the length of the seizure and the time point (t1) beyond which the seizure should be regarded as ‘continuous seizure activity’*” (11, 17). Hitherto, the SE in animal models has been defined as the duration from the onset of seizures until they stop on their own or intervention by an AED, most commonly diazepam (DZP). This means the length of behavioral seizures, i.e., SE could vary between animals. This can cause problems in animals that are intended for interventional and long-term studies (for example, vehicle treated versus test drug treated group/s), and would yield confounding results. Since DZP has little or no effect on EEG seizures, it is unlikely to have an impact on brain pathology in animals that experience severe SE (49, 50). However, DZP treatment controlled behavioral seizures and suppressed epileptiform spikes or electrographic seizures in mice that had mild SE (45). According to the NIH CounterACT program, administering DZP or other AEDs to control behavioral seizures prior to testing disease-modifying agent is recommended for translational studies. The length of SE also depends on the type of chemoconvulsant and the method of administration. For example, SHD method of kainate (i.p.) produces inconsistent seizure response with high variability in SE duration, while the RLD method of administration (i.p.) produces relatively consistent SE (stage ≥3) with longer duration in both mice and rats (2, 9). Therefore, the RLD method is useful to develop animal models of varying SE severity, such as mild or severe groups, for any chosen experiment.

Several studies have shown huge variations in SE response to a SHD of kainate in various strains of mice and rats in terms of SE onset and duration (2, 23, 43, 51, 52). Unlike in humans, the convulsive SE is not always continuous in animal models, and the seizures fluctuate either between stage 1 and stage 5 or in between the stages of CS (stages 3 and 5). During continuous SE, the electrographic activity does not always correspond to the behavioral seizures. Furthermore, progression of NCS to CS is not always consistent in mice and rats. For example, during the transition from NCS to CS, we observed a pattern of HFT spikes on the EEG that had no behavioral correlation in both mice and rats (Figures 1A,B). During SE, a variety of epileptiform spiking activities were observed on the EEG, even though the animals were not exhibiting any behavioral seizures. These include spike trains containing isolated epileptiform spikes or spike clusters with <12-s duration (Figures 2B–E) (45). Since not every experiment will require telemetry, a video acquisition, in addition to direct observation, is useful to quantify the exact duration of convulsive SE in a given time. To precisely quantify the severity of SE, we considered the exact duration of CS (stage ≥3) that occurred between the first onset of CS following kainate injection and the time the DZP was administered. By RLD of kainate, we could achieve >30 min of continuous CS in 95% of the animals with <15% mortality rate in both mice (irrespective of strains) and rats. These results are consistent with the previous studies (2, 43, 44, 51, 53). Irrespective of the model, DZP at 10 mg/kg (i.p.) terminated behavioral seizures in the vast majority of animals, but had little or no impact on continuous seizure activity, i.e., epileptiform spiking activity in those animals that experienced severe seizures (45, 54–56). However, DZP reduced mortality rate in animals with severe SE to some extent (56–58), but completely suppressed epileptiform spikes in animals that had mild SE (45). It has been known that severe seizures cause either internalization or inactivation of GABA_A_ receptors at the synaptic terminals that result in diminished response to the DZP treatment (8, 49, 54, 59, 60).

In summary, the length of the convulsive SE in animal models usually lasts for >30 min by the RLD method of kainate, in contrast to a SHD method. The severity of SE can be determined by quantifying the exact duration of different stages of CS (stages 3–5) from continuous video recording and/or by direct observation. Telemetry implanted animals require ~40% less kainate to induce convulsive SE. The epileptiform spike frequency can be considered to determine the severity of SE in addition to behavioral seizures quantification. This approach will reduce variability in determining the initial severity of convulsive SE in disease-modifying experiments that require long-term video-EEG monitoring. In addition to the power spectral analysis, it may be useful to quantify stage-specific spike frequencies during SE to determine the severity of SE and to correlate with behavioral seizures where appropriate. The refined RLD method of kainate administration overcomes some of the shortcomings in generating convulsive SE models, reduces mortality rate, and the cost of experiments.

## Author Contributions

SS: performed the experiments, analyzed the data, reviewed the literature, wrote the manuscript, and prepared the tables and figures. SP: performed the experiments and analyzed the data; AK: contributed animals. TT: conceived the idea, designed the study, and wrote/edited the manuscript.

## Conflict of Interest Statement

The authors declare that the research was conducted in the absence of any commercial or financial relationships that could be construed as a potential conflict of interest.
